# Epidemiology of pediatric trauma during the COVID-19 pandemic shelter in place^☆^

**DOI:** 10.1016/j.sopen.2021.06.001

**Published:** 2021-06-22

**Authors:** Kovi E. Bessoff, Ryan W. Han, Min Cho, Melanie Stroud, Eva M. Urrechaga, Chad M. Thorson, Katie W. Russell, Autumn Rohan, Shannon N. Acker, Shakeva Swain, Leopoldo Malvezzi, Julie R. Fuchs, Stephanie D. Chao

**Affiliations:** a Stanford School of Medicine, Department of Surgery, Stanford, CA; b Stanford University, Department of Computer Science, Stanford, CA; c Stanford University, Department of Statistics, Stanford, CA; d Lucile Packard Children's Hospital Stanford, Palo Alto, CA; e University of Miami/Miller School of Medicine, Department of Surgery, Miami, FL; f University of Miami/Miller School of Medicine, Department of Surgery, Division of Pediatric Surgery, Miami, FL; g University of Utah School of Medicine, Department of Surgery, Division of Pediatric Surgery, Salt Lake City, UT; h Children's Hospital Colorado, Division of Pediatric Surgery, University of Colorado School of Medicine, Aurora, CO; i Nicklaus Children's Hospital, Department of Trauma Services, Miami, FL; j Nicklaus Children's Hospital, Miami Associates in Pediatric Surgery, Department of Pediatric Surgery, Miami, FL; k Santa Clara Valley Medical Center, Department of Surgery, San Jose, CA; l Stanford School of Medicine, Department of Surgery, Division of Pediatric Surgery, Stanford, CA

## Abstract

**Introduction:**

The first COVID-19 cases occurred in the US in January of 2020, leading to the implementation of shelter in place. This study seeks to define the impact of shelter in place on the epidemiology of pediatric trauma.

**Methods:**

We examined pediatric trauma admissions at 5 Level 1 and 1 Level 2 US pediatric trauma centers between January 1 and June 30, 2017–2020. Demographic and injury data were compared between pre– and post–shelter in place patient cohorts.

**Results:**

A total of 8772 pediatric trauma activations were reviewed. There was a 13% decrease in trauma volume in 2020, with a nadir at 16 days following implementation of shelter in place. Injury severity scores were higher in the post–shelter in place cohort. The incidence of nonmotorized vehicle accidents and gunshot wounds increased in the post–shelter in place cohort.

**Conclusion:**

We found an overall decrease in pediatric trauma volume following shelter in place. However, injuries tended to be more severe. Our findings help inform targeted injury prevention campaigns during future pandemics.

## Highlights

• Shelter-in-place policies led to a 13% decrease in overall pediatric trauma volume.
• Biggest decrease occurred in the 30 days surrounding the start of shelter in place.
• Nonmotorized vehicle accidents and gunshot wounds increased with shelter in place.
• Injury severity was higher among the shelter-in-place cohort compared to controls.
• The incidence of nonaccidental trauma did not change with shelter in place.

## BACKGROUND

The first human COVID-19 infections occurred in Wuhan, China, in December of 2019 [1], and the first case of the disease was confirmed in the US on January 20, 2020 [2]. Subsequent cases were reported in Santa Clara County, California; Douglas County, Colorado; Davis County, Utah; and Miami-Dade County, Florida, in close succession, illustrating the virus' penchant for rapid spread. Public health interventions, including issuance of shelter in place (SIP) orders, were implemented in these counties (Table 1) in an effort to dampen viral transmission.

**Table 1:** Description of participating pediatric trauma centers and SIP dates

*Name of center*	*Location (city, state)*	*County*	*Pediatric trauma level designation*	*Date of state's first case (county)*	*Start date: shelter in place*	*End date: shelter in place*
Nicklaus Children's Hospital	Miami, FL	Miami-Dade	1	3/11/2020 (Miami-Dade)	3/24/2020	5/18/2020
Jackson Memorial Hospital/Ryder Trauma Center	Miami, FL	Miami-Dade	1	3/11/2020 (Miami-Dade)	3/24/2020	5/18/2020
Children's Hospital Colorado	Aurora, CO	Adams	1	3/5/2020 (Douglas)	3/25/2020	5/8/2020
Lucile Packard Children's Hospital	Palo Alto, CA	Santa Clara	1	2/20/2020 (Santa Clara)	3/16/2020	5/25/2020
Santa Clara Valley Medical Center	San Jose, CA	Santa Clara	2	2/20/2020 (Santa Clara)	3/16/2020	5/25/2020
Primary Children's Hospital	Utah	Salt Lake	1	3/6/2020 (Davis)	3/30/2020	5/1/2020

Data suggest that SIP had a significant impact on hospital utilization. Examination of over 1,000,000 hospital admissions revealed a 43% decline in non-COVID medical admissions from January to April 2020 [3], and the Centers for Disease Control and Prevention (CDC) noted a 70% decrease in emergency department visits for patients ≤ 14 years of age during the same time period [4]. Single-center retrospective reviews during this time period have shown similar declines in the rate of adult trauma [5,6] despite increased injury severity [6]. Although reports are sparse, similar trends have also been noted in pediatric orthopedic trauma in the United States [7] and abroad [8,9].

In addition to changes in trauma volume, many experts anticipated differences in the etiology of pediatric trauma following the implementation of SIP. Increased stress associated with the pandemic, strained relationships, and social isolation were postulated to contribute to an increase in domestic violence including nonaccidental trauma (NAT) in children [10,11]. These risk factors were compounded by the fact that vulnerable children were isolated from the limited resources they had, including schools and community centers [12]. Additionally, the rate of firearms sales increased substantially with the onset of the pandemic [13], creating additional risk factors for accidental and nonaccidental traumatic incidents. Early reports show an increase in gun-related trauma in adults following the implementation of SIP [14]. With this in mind, we sought to characterize the impact of COVID-19 SIP orders on pediatric trauma during the early months of the pandemic. Based on previous trends, we hypothesized that overall trauma volume would decrease but expected to see a higher volume of domestic violence, gun violence, and NAT.

## METHODS

Pediatric trauma admissions from 5 Level 1 and 1 Level 2 pediatric trauma centers (Table 1) in 4 US states (California, Utah, Colorado, and Florida) were reviewed. Patients ≤ 18 years old who met trauma registry criteria from January 1 to June 30, 2017–2020, were compiled by each institution. Data were deidentified and pooled for analysis relative to their county's SIP order.

Demographic and injury data from patients injured in 2020 before (pre-SIP cohort) and after SIP (post-SIP cohort) were compared to date-matched controls (controls) from 2017 to 2019. Supplemental file 1 describes the variables examined. The Wilcoxon rank-sum test was used to compare numerical variables, and the Fisher exact test was used to compare incidence and volume distribution between years. Bonferroni corrections were applied where multiple comparisons were made. Analysis was performed using R version 4.0.0 (R Foundation for Statistical Computing, Vienna, Austria). IRB approval was obtained from each center.

## RESULTS

**Fig 1:**
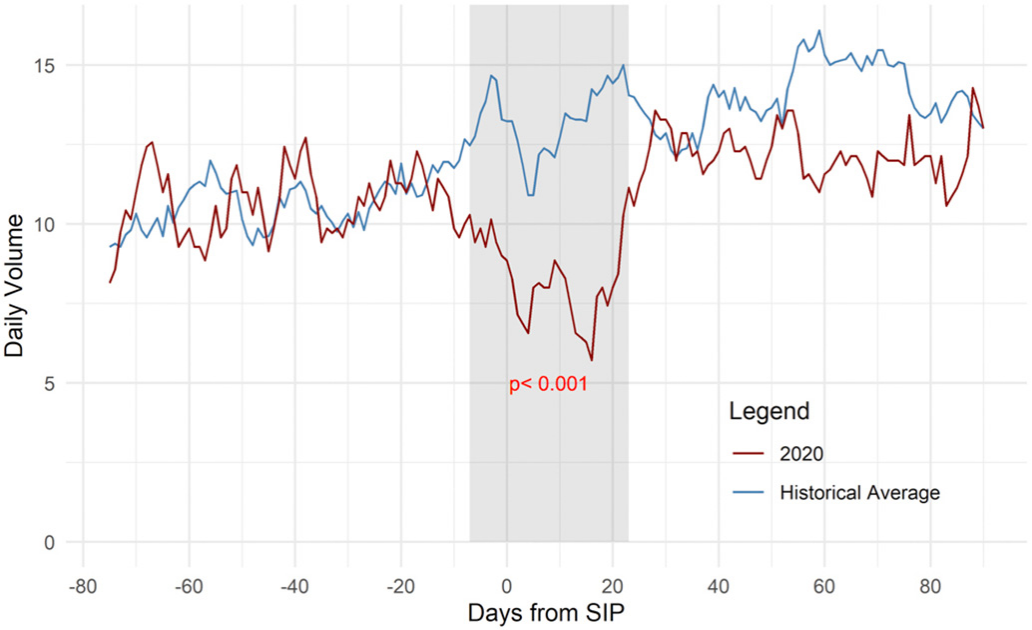
Daily trauma volume aggregated from 6 pediatric trauma centers across the US from January to June, 2017–2020. Dates on the horizontal axis were normalized relative to the shelter-in-place order for each center's county; trauma volume was plotted using a 7-day running average. The shaded area marks the 30-day period which saw the most significant change in pediatric trauma volume. *P* value was calculated using Fisher exact test to compare volume in 2020 to historical controls.

**Fig 2:**
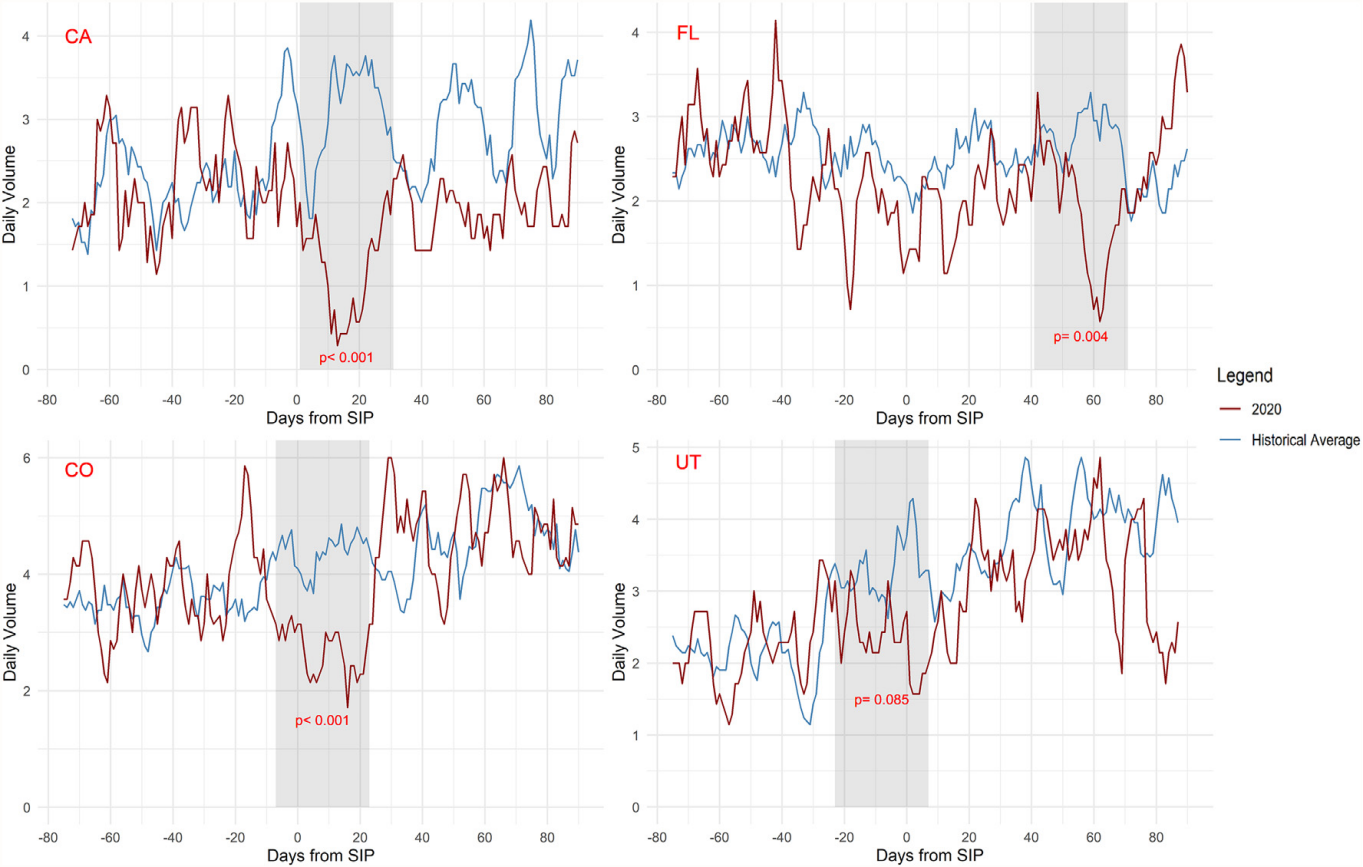
Daily trauma volume by state for 6 pediatric trauma centers across the US from January to June, 2017–2020. Dates on the horizontal axis were normalized relative to the shelter-in-place order for each center's county; trauma volume was plotted using a 7-day running average. The shaded areas mark the 30-day periods which saw the most significant change in pediatric trauma volume for each state. *P* value was calculated using Fisher exact test to compare volume in 2020 to historical controls.

A total of 1972 trauma activations occurred in 2020, 2277 in 2017, 2244 in 2018, and 2279 in 2019, representing an approximately 13% reduction in trauma volume. Figure 1 shows decreased cumulative trauma volumes across all centers starting approximately 20 days prior to SIP compared to historical controls. The 30 days surrounding SIP (7 days before through 22 days after) saw the greatest change in volumes across all centers (*P* < .001). Daily mean trauma volume 16 days after SIP saw the greatest decline at a 59.9% decrease from the historical average (5.7 vs 14.2 patients). Figure 2 shows differences in trauma volumes by state. Shaded boxes highlight the 30-day window with the most significant change in trauma volumes. In California (9 days pre-SIP to 20 days post-SIP) and Colorado (11 days pre-SIP to 18 days post-SIP), this window occurred around the implementation, whereas it did not occur until much later in Florida (41 days post-SIP to 70 days post-SIP). Trauma volumes in Utah did not decrease significantly (*P* = .085).

Race, ethnicity, age, sex, arrival time (Table 2), insurance type, median income by zip code, and day of the week (data not shown) did not differ between the pre-SIP cohort and controls when examined across all centers. Following SIP, trauma volume decreased between 8:00 and 15:00 and increased between 18:00 and 22:00. This change appears to be driven by the Colorado cohort (Supplemental Table 2). Other demographic data did not vary when examined across all centers (Table 2). When demographic data were examined by state (Supplemental Table 2), the proportion of patients covered by government insurance programs increased in California (48.7% vs 39.7%, *P* = .027) in the post-SIP cohort compared to controls. Florida saw significantly more Hispanic patients (60.9% vs 49.9%, *P* = .008) in the post-SIP cohort compared to controls. Finally, age increased in the Colorado cohort (7 [4, 12] years old vs 6 [3, 11] years old, *P* = .012) and decreased in the Florida cohort (6 [3, 14] vs 9 [4, 15], *P* = .006) in the post-SIP cohort compared to controls.

Mechanism of injury did not differ between the pre-SIP cohort and controls (overall *P* = .051). Following SIP, the incidence of nonmotorized vehicle accidents (15.0% vs 8.9%, *P* < .001) and gunshot wounds (3.3% vs 1.8%, *P* = .002) increased compared to controls across all centers (Table 3). There was a significant change in the admitting service for trauma patients post-SIP compared to age-matched controls (overall *P* = .026), which may further illustrate changes in the mechanism and type of injuries suffered by patients post-SIP. Specifically, the number of admissions to facial surgery services decreased (13.1% in 2017–2019 cohort vs 10.6% in 2020 cohort, *P* = .03) (Table 2).

**Table 2:** Demographic descriptions of pre-SIP and post-SIP cohorts

	*Pre-SIP*			*Post-SIP*		
	*2017–2019*	*2020*	P *value*	*2017–2019*	*2020*	P *value*
	*(*N *= 2758)*	*(*N *= 882)*		*(*N *= 4042)*	*(*N *= 1090)*	
Race						
Asian/Pacific Islander	111 (4.0%)	37 (4.2%)	.336	197 (4.9%)	49 (4.5%)	.324
Black	237 (8.6%)	77 (8.7%)		335 (8.3%)	76 (7.0%)	
Native American	18 (0.7%)	6 (0.7%)		41 (1.0%)	5 (0.5%)	
Other	366 (13.3%)	89 (10.1%)		580 (14.3%)	152 (13.9%)	
White	1924 (69.8%)	597 (67.7%)		2763 (68.4%)	748 (68.6%)	
Missing	102 (3.7%)	76 (8.6%)		126 (3.1%)	60 (5.5%)	

Ethnicity						
Hispanic	932 (33.8%)	308 (34.9%)	.234	1310 (32.4%)	363 (33.3%)	.189
Not Hispanic	1745 (63.3%)	523 (59.3%)		2619 (64.8%)	659 (60.5%)	
Missing	81 (2.9%)	51 (5.8%)		113 (2.8%)	68 (6.2%)	

Age						
Median [Q1, Q3]	8 [3, 13]	9 [4, 14]	.128	8 [3, 13]	7 [3, 13]	.857

Sex						
Female	995 (36.1%)	325 (36.8%)	.705	1561 (38.6%)	407 (37.3%)	.465
Male	1763 (63.9%)	557 (63.2%)		2481 (61.4%)	683 (62.7%)	

Arrival time						
8:00–15:00	583 (21.1%)	176 (20.0%)	.817	824 (20.4%)	179 (16.4%)	.002⁎
15:00–18:00	543 (19.7%)	184 (20.9%)		764 (18.9%)	207 (19.0%)	
18:00–22:00	864 (31.3%)	280 (31.7%)		1206 (29.8%)	392 (36.0%)	
22:00–8:00	727 (26.4%)	236 (26.8%)		1188 (29.4%)	310 (28.4%)	
Missing	41 (1.5%)	6 (0.7%)		60 (1.5%)	2 (0.2%)	

ISS						
Median [Q1, Q3]	4 [4, 9]	4 [4, 9]	.656	4 [3, 9]	5 [4, 10]	<.001⁎
Missing	97 (3.5%)	32 (3.6%)		147 (3.6%)	36 (3.3%)	

ICU length of stay						
Median [Q1, Q3]	2 [0, 3]	1 [0, 3]	.075	1 [0, 3]	2 [1, 3]	<.001⁎
Missing	2140 (77.6%)	701 (79.5%)		3078 (76.2%)	844 (77.4%)	

ICU admission						
Yes	330	95	.336	517	157	.162
No	2428	787		3525	933	

Admitting service						
Critical care	113 (4.1%)	33 (3.7%)	.761	144 (3.6%)	34 (3.1%)	.026⁎
Face	333 (12.1%)	86 (9.8%)		531 (13.1%)	116 (10.6%)	
General surgery	953 (34.6%)	317 (35.9%)		1380 (34.1%)	442 (40.6%)	
GU	11 (0.4%)	2 (0.2%)		18 (0.4%)	4 (0.4%)	
Hand	6 (0.2%)	2 (0.2%)		11 (0.3%)	2 (0.2%)	
Neuro	94 (3.4%)	26 (2.9%)		159 (3.9%)	44 (4.0%)	
Nonsurgical	124 (4.5%)	35 (4.0%)		149 (3.7%)	36 (3.3%)	
Orthopedic surgery	421 (15.3%)	140 (15.9%)		591 (14.6%)	170 (15.6%)	
Plastic surgery	36 (1.3%)	11 (1.2%)		48 (1.2%)	22 (2.0%)	
Missing	667 (24.2%)	230 (26.1%)		1010 (25.0%)	219 (20.1%)	

⁎ Statistically significant difference between 2020 and the historical average.

**Table 3:** Mechanism of injury post-SIP compared to date-matched controls⁎

	*2017–2019*	*2020*	P *value*
	*(*N *= 4042)*	*(*N *= 1090)*	
Mechanism of injury			
Animal	127 (3.1%)	44 (4.0%)	1
Assault	38 (0.9%)	16 (1.5%)	1
Blunt	376 (9.3%)	80 (7.3%)	.815
Crush	40 (1.0%)	12 (1.1%)	1
Exposure/burn	156 (3.9%)	40 (3.7%)	1
Fall	1630 (40.3%)	400 (36.7%)	.637
Gunshot	71 (1.8%)	36 (3.3%)	.038
Stab/lacerations	68 (1.7%)	24 (2.2%)	1
Motorized vehicle	817 (20.2%)	201 (18.4%)	1
Nonaccidental trauma	94 (2.3%)	21 (1.9%)	1
Nonmotorized vehicle	360 (8.9%)	163 (15.0%)	<.001
Pedestrian	171 (4.2%)	27 (2.5%)	.1134
Unknown/other	75 (1.9%)	16 (1.5%)	1
Missing	19 (0.5%)	9 (0.8%)	

⁎ Statistically significant difference between 2020 and the historical average.

Acuity of injury (injury severity score [ISS]), need for ICU admission, and ICU length of stay (LOS) between the pre-SIP cohort and controls did not differ. Following SIP, median ISSs were higher compared to controls (5 [4, 10] vs 4 [3, 9]; *P* < .01) (Table 2). The percentage of patients with severe injuries (ISS ≥ 25) [15] increased during SIP (5.8% vs 3.5% pre-SIP, *P* = .019). This trend was not present during control years (4.6% April–June vs 5.1% January–March; *P* = .30). Likelihood of ICU admission did not differ, but median ICU LOS was higher in post-SIP patients compared to controls (Table 2). Pediatric trauma mortality following SIP did not differ compared to controls (1.3% vs 1.2%, *P* = .76).

## DISCUSSION

This study examines the impact of the COVID-19 pandemic on the epidemiology of pediatric trauma across 6 centers in 4 states and is one of the largest studies of pediatric trauma etiology during this period. We noted a significant decrease in the incidence of pediatric trauma immediately following the implementation of SIP. Although data regarding the incidence of nonorthopedic pediatric trauma in the early phase of the COVID-19 pandemic is sparse, these findings correlate with findings in the adult trauma population [5,16,17]. Work by Bram and colleagues on pediatric fractures also noted over a 2-fold decrease in fracture volume during the pandemic. Additionally, they found an increased rate of bicycle injuries during lockdown compared to controls, similar to our findings [7]. Proposed reasons for this change include a shift from organized sports or playground activities toward an increase in children spending time doing more "pandemic-appropriate" activities, including playing at home and outdoors in their neighborhoods.

Patients who did present to the hospital had higher ISS, more severe injuries, and longer ICU stays, suggesting that those who sought care suffered more significant trauma, a phenomenon noted in another work [6]. We also noted a shift in the mechanisms responsible for trauma activations during the lockdown. Penetrating trauma, and gunshot wounds in particular, increased in the post-SIP cohort. There was a nearly 2-fold increase in the incidence of penetrating trauma. These findings corroborate work by Abdallah et al [14] and are concerning given the increasing prevalence of firearms in homes [13]. Similarly, a commentary out of Philadelphia by Hatchmonji and colleagues described an increase in firearm violence across their community early in the lockdown [18]. Reasons proposed by the group included the increase in firearm sales as we noted previously, as well as a potential increased risk of community violence due to the stay-at-home orders in disadvantaged neighborhoods [18].

Despite widely held concerns that SIP would increase the incidence of domestic violence and child abuse, our work did not find a significance difference in the incidence of NAT between the pre-SIP and post-SIP cohorts. This was unanticipated and is in contrast to work by Kovler et al [19] that revealed a 2-fold increase in NAT at a single Level 1 pediatric trauma center. However, work from the UK and Ireland has had similar findings to ours, with no observable increase in NAT [9,20]. Nevertheless, close observations of trends in NAT remain imperative, as increasing pressure in the home from prolonged lockdowns may ultimately drive rates up as the pandemic continues.

The major weakness of the study is its retrospective nature. However, data were collected using prospectively maintained trauma registries. The goal of this study was to examine the specific effect of SIP on pediatric trauma trends. Further studies examining a longer time period may provide additional insight into the effect of a pandemic on pediatric injuries. Strengths include a large, geographically and socioeconomically diverse patient population.

Our study found that although overall trauma volume decreased around SIP, the nature of traumatic injuries tended to be more severe after SIP. Overall, we found an increase in nonmotorized vehicle accidents (eg, bicycle accidents) and penetrating trauma (eg, firearm injury). Our findings may help inform targeted injury prevention campaigns during future surges of COVID-19. Lockdowns in vulnerable neighborhoods must be coupled with increased social support to discourage further violent injury. Education on appropriate safety measures, such as helmets and protective gear, is essential for children increasingly riding their bicycles in the neighborhood.

The following are the supplementary data related to this article.

Supplemental Tables Supplemental Tables

Supplemental Table 2 Post-SIP cohort demographics by state Supplemental Table 2

## Author Contribution

Dr Bessoff and Mr. Han prepared the manuscript, contributed to study conceptualization and design, designed data collection instruments, and performed data analysis.

Dr Chao, Ms Stroud, and Ms Cho contributed to study conceptualization and design, designed the data collection instruments, performed data analysis, and provided critical review of the manuscript including revisions.

Dr Urrechaga, Dr Thorson, Dr Russell, Dr Acker, Dr Malvezzi, Dr Fuchs, Ms Rohan, and Ms Swain coordinated and supervised data collection at their corresponding institutions and provided critical review of the manuscript including revisions.

All authors approved the final manuscript as submitted and agree to be accountable for all aspects of the work.

## Conflict of Interest

The authors have no conflicts of interest relevant to this article to disclose.

## Funding Source

This project was completed with no specific support.
