# Supplementary material for: Epidemiology of pediatric trauma during the COVID-19 pandemic shelter in place
Source: Surg Open Sci. 2021 Jun 22;6:5–9. doi: 10.1016/j.sopen.2021.06.001 (PMC8275016; doi:10.1016/j.sopen.2021.06.001)
Supplement: Supplementary file 1 — Supplemental Tables [file mmc1.docx]

Supplemental table 1:

| **VARIABLE** | **EXPLANATION** |
| --- | --- |
| **Cause of Injury** |  |
| Gunshot wounds | High velocity penetrating wounds (gunshot wounds) |
| Stab/laceration | Low velocity penetrating wounds including stab wounds and lacerations |
| Crush | crush injury |
| Blunt | Blunt trauma, excluding assault and non-accidental trauma |
| Assault | assault, exclusive of non-accidental trauma |
| Non accidental trauma | suspected or confirmed non-accidental trauma |
| Fall | fall from any height, including into water |
| Exposure/burn | exposure or burn |
| Motorized vehicle | any injury involving a motorized vehicle other than pedestrian struck |
| Non-motorized vehicle | any injury involving a non-motorized conveyance |
| Pedestrian | pedestrian vs. motorized vehicle |
| Animal | any injury involving an animal attack or bite or if patients was thrown/fell from or stepped on by an animal |
| Foreign body | Ingestion of a foreign body |
| Unknown/other | unknown mechanism of injury, or mechanism of injury not otherwise specified |
| **Activation levels** |  |
| Major | Tier 1 activation |
| Minor | Tier 2 activation |
| Consult | Patients with minor injuries not requiring trauma activation |
| **Emergency department disposition** |  |
| Discharged | Discharged to home, jail, social services, mental health services. Includes leaving AMA |
| Floor | Admitted to floor with surgical service, pediatric service, observation, telemetry floor, or step down |
| Operating room | Patient taken directly to OR from trauma bay |
| ICU | Includes surgical ICU, pediatric ICU, and neonatal ICU |
| Transferred | transferred to other acute care hospital hospital |
| Died | Patient death called in the trauma bay |
| **Hospital discharge disposition** |  |
| Died | Patient died during index hospitalization |
| Rehab/skilled nursing | Includes skilled nursing facility, long term acute care hospital, acute rehabilitation unit, transitional care unit, or other rehabilitation unit |
| Discharged | includes home health, discharge AMA, home with services, discharge to child protective services/foster care, discharge to law enforcement/jail, psychiatric facility |
| Transferred | Transferred to another acute care hospital |
| **Admitting service** |  |
| General surgery | includes trauma and pediatric surgery services |
| Neuro | includes neurosurgery, orthospine, and neurology services |
| Critical care | includes pediatric intensivist, critical care, ICU, neonatology, and burn services |
| Non-surgical | includes general pediatrics, emergency medicine, medicine, cardiology, and gastroenterology |
| Face | includes dental, otolaryngology, and ophthalmology services |
| Hand | includes dedicated hand service, as well as plastic surgery and orthopedic surgery when designated as the covering hand team |
| GU | includes urology and OB/GYN services |
| Orthopedic surgery |  |
| Plastic surgery |  |
| Vascular | includes vascular surgery and reimplantation services |
