# Supplementary material for: Epidemiology of pediatric trauma during the COVID-19 pandemic shelter in place
Source: Surg Open Sci. 2021 Jun 22;6:5–9. doi: 10.1016/j.sopen.2021.06.001 (PMC8275016; doi:10.1016/j.sopen.2021.06.001)
Supplement: Supplemental Table 2 — Post-SIP cohort demographics by state [file mmc2.docx]

**Supplemental Table 2:** Post-SIP cohort demographics by state

|  | **CA** | |  | **CO** | |  | **FL** | |  | **UT** | |  |
| --- | --- | --- | --- | --- | --- | --- | --- | --- | --- | --- | --- | --- |
|  | **2017-2019** | **2020** | **p-value** | **2017-2019** | **2020** | **p-value** | **2017-2019** | **2020** | **p-value** | **2017-2019** | **2020** | **p-value** |
|  | **(N=942)** | **(N=191)** |  | **(N=1325)** | **(N=409)** |  | **(N=742)** | **(N=207)** |  | **(N=1033)** | **(N=283)** |  |
| **Race** |  |  |  |  |  |  |  |  |  |  |  |  |
| Asian/Pacific Islander | 131 (13.9%) | 37 (19.4%) | 0.286 | 32 (2.4%) | 4 (1.0%) | 0.158 | 2 (0.3%) | 0 (0%) | 0.305 | 32 (3.1%) | 8 (2.8%) | 0.958 |
| Black | 34 (3.6%) | 4 (2.1%) |  | 69 (5.2%) | 19 (4.6%) |  | 214 (28.8%) | 49 (23.7%) |  | 18 (1.7%) | 4 (1.4%) |  |
| Native American | 3 (0.3%) | 0 (0%) |  | 18 (1.4%) | 2 (0.5%) |  | 2 (0.3%) | 0 (0%) |  | 18 (1.7%) | 3 (1.1%) |  |
| Other | 231 (24.5%) | 50 (26.2%) |  | 298 (22.5%) | 81 (19.8%) |  | 51 (6.9%) | 21 (10.1%) |  | 0 (0%) | 0 (0%) |  |
| White | 538 (57.1%) | 100 (52.4%) |  | 858 (64.8%) | 279 (68.2%) |  | 473 (63.7%) | 136 (65.7%) |  | 894 (86.5%) | 233 (82.3%) |  |
| Missing | 5 (0.5%) | 0 (0%) |  | 50 (3.8%) | 24 (5.9%) |  | 0 (0%) | 1 (0.5%) |  | 71 (6.9%) | 35 (12.4%) |  |
| **Ethnicity** |  |  |  |  |  |  |  |  |  |  |  |  |
| Hispanic | 432 (45.9%) | 82 (42.9%) | 0.427 | 362 (27.3%) | 115 (28.1%) | 0.494 | 370 (49.9%) | 126 (60.9%) | 0.008* | 146 (14.1%) | 40 (14.1%) | 0.608 |
| Not Hispanic | 504 (53.5%) | 109 (57.1%) |  | 909 (68.6%) | 262 (64.1%) |  | 372 (50.1%) | 81 (39.1%) |  | 834 (80.7%) | 207 (73.1%) |  |
| Missing | 6 (0.6%) | 0 (0%) |  | 54 (4.1%) | 32 (7.8%) |  | 0 (0%) | 0 (0%) |  | 53 (5.1%) | 36 (12.7%) |  |
| **Age** |  |  |  |  |  |  |  |  |  |  |  |  |
| Median [Q1, Q3] | 8 [3, 14] | 8 [3, 13] | 0.378 | 6 [3, 11] | 7 [4, 12] | 0.012* | 9 [4, 15] | 6 [3, 14] | 0.006* | 8 [4, 12] | 8 [4, 13] | 0.569 |
| **Sex** |  |  |  |  |  |  |  |  |  |  |  |  |
| Female | 380 (40.3%) | 74 (38.7%) | 0.730 | 500 (37.7%) | 154 (37.7%) | 1 | 276 (37.2%) | 69 (33.3%) | 0.361 | 405 (39.2%) | 110 (38.9%) | 0.953 |
| Male | 562 (59.7%) | 117 (61.3%) |  | 825 (62.3%) | 255 (62.3%) |  | 466 (62.8%) | 138 (66.7%) |  | 628 (60.8%) | 173 (61.1%) |  |
| **Payment_Source** |  |  |  |  |  |  |  |  |  |  |  |  |
| Government | 374 (39.7%) | 93 (48.7%) | 0.027* | 637 (48.1%) | 197 (48.2%) | 0.440 | 338 (45.6%) | 91 (44.0%) | 0.831 | 226 (21.9%) | 65 (23.0%) | 0.603 |
| Private | 530 (56.3%) | 85 (44.5%) |  | 623 (47.0%) | 185 (45.2%) |  | 342 (46.1%) | 101 (48.8%) |  | 708 (68.5%) | 198 (70.0%) |  |
| Self-pay | 34 (3.6%) | 6 (3.1%) |  | 65 (4.9%) | 26 (6.4%) |  | 57 (7.7%) | 15 (7.2%) |  | 92 (8.9%) | 20 (7.1%) |  |
| Missing | 4 (0.4%) | 7 (3.7%) |  | 0 (0%) | 1 (0.2%) |  | 5 (0.7%) | 0 (0%) |  | 7 (0.7%) | 0 (0%) |  |
| **Median Zipcode Income Quartile** |  |  |  |  |  |  |  |  |  |  |  |  |
| $1 under $25,000 | 2 (0.2%) | 0 (0%) | 0.304 | 2 (0.2%) | 1 (0.2%) | 0.472 | 327 (44.1%) | 104 (50.2%) | 0.852 | 9 (0.9%) | 2 (0.7%) | 0.810 |
| $25,000 under $50,000 | 408 (43.3%) | 76 (39.8%) |  | 813 (61.4%) | 236 (57.7%) |  | 268 (36.1%) | 82 (39.6%) |  | 574 (55.6%) | 151 (53.4%) |  |
| $50,000 under $75,000 | 256 (27.2%) | 44 (23.0%) |  | 296 (22.3%) | 104 (25.4%) |  | 28 (3.8%) | 9 (4.3%) |  | 311 (30.1%) | 92 (32.5%) |  |
| $75,000 under $100,000 | 89 (9.4%) | 23 (12.0%) |  | 137 (10.3%) | 49 (12.0%) |  | 1 (0.1%) | 0 (0%) |  | 2 (0.2%) | 1 (0.4%) |  |
| $100,000 under $200,000 | 168 (17.8%) | 43 (22.5%) |  | 15 (1.1%) | 5 (1.2%) |  | 10 (1.3%) | 5 (2.4%) |  | 2 (0.2%) | 0 (0%) |  |
| Missing | 19 (2.0%) | 5 (2.6%) |  | 62 (4.7%) | 14 (3.4%) |  | 108 (14.6%) | 7 (3.4%) |  | 135 (13.1%) | 37 (13.1%) |  |
| **Weekday** |  |  |  |  |  |  |  |  |  |  |  |  |
| Yes | 620 (65.8%) | 130 (68.1%) | 0.599 | 904 (68.2%) | 266 (65.0%) | 0.213 | 513 (69.1%) | 143 (69.1%) | 1 | 688 (66.6%) | 182 (64.3%) | 0.491 |
| No | 322 (34.2%) | 61 (31.9%) |  | 421 (31.8%) | 143 (35.0%) |  | 229 (30.9%) | 64 (30.9%) |  | 345 (33.4%) | 101 (35.7%) |  |
| **Arr_Time_Group** |  |  |  |  |  |  |  |  |  |  |  |  |
| 8:00 - 15:00 | 235 (24.9%) | 34 (17.8%) | 0.066 | 223 (16.8%) | 60 (14.7%) | 0.035* | 163 (22.0%) | 39 (18.8%) | 0.618 | 203 (19.7%) | 46 (16.3%) | 0.415 |
| 15:00 - 18:00 | 188 (20.0%) | 45 (23.6%) |  | 243 (18.3%) | 76 (18.6%) |  | 134 (18.1%) | 36 (17.4%) |  | 199 (19.3%) | 50 (17.7%) |  |
| 18:00 - 22:00 | 246 (26.1%) | 64 (33.5%) |  | 388 (29.3%) | 149 (36.4%) |  | 225 (30.3%) | 72 (34.8%) |  | 347 (33.6%) | 107 (37.8%) |  |
| 22:00 - 8:00 | 213 (22.6%) | 46 (24.1%) |  | 471 (35.5%) | 124 (30.3%) |  | 220 (29.6%) | 60 (29.0%) |  | 284 (27.5%) | 80 (28.3%) |  |
| Missing | 60 (6.4%) | 2 (1.0%) |  | 0 (0%) | 0 (0%) |  | 0 (0%) | 0 (0%) |  | 0 (0%) | 0 (0%) |  |
| **Activation Level** |  |  |  |  |  |  |  |  |  |  |  |  |
| Tier 1 | 95 (10.1%) | 13 (6.8%) | 0.410 | 71 (5.4%) | 19 (4.6%) | 0.272 | 43 (5.8%) | 10 (4.8%) | 0.003* | 108 (10.5%) | 38 (13.4%) | 0.392 |
| Tier 2 | 571 (60.6%) | 108 (56.5%) |  | 51 (3.8%) | 21 (5.1%) |  | 10 (1.3%) | 13 (6.3%) |  | 177 (17.1%) | 49 (17.3%) |  |
| Data not available | 276 (29.3%) | 70 (36.6%) |  | 1203 (90.8%) | 369 (90.2%) |  | 689 (92.9%) | 184 (88.9%) |  | 748 (72.4%) | 196 (69.3%) |  |
| **ISS** |  |  |  |  |  |  |  |  |  |  |  |  |
| Median [Q1, Q3] | 4 [1, 9] | 4 [2, 9] | 0.003* | 4 [4, 9] | 4 [4, 9] | 0.082 | 4 [1, 9] | 4 [1, 9] | 0.132 | 5 [4, 10] | 9 [4, 10] | 0.002* |
| Missing | 121 (12.8%) | 27 (14.1%) |  | 14 (1.1%) | 4 (1.0%) |  | 1 (0.1%) | 0 (0%) |  | 11 (1.1%) | 5 (1.8%) |  |
| **Hospital Length of Stay** |  |  |  |  |  |  |  |  |  |  |  |  |
| Median [Q1, Q3] | 1 [1, 2] | 1 [1, 2] | 0.040* | 1 [1, 2] | 1 [1, 2] | 0.539 | 2 [2, 4] | 2 [2, 3] | 0.133 | 1 [1, 2] | 1 [1, 2] | 0.356 |
| Missing | 1 (0.1%) | 0 (0%) |  | 153 (11.5%) | 48 (11.7%) |  | 87 (11.7%) | 15 (7.2%) |  | 11 (1.1%) | 1 (0.4%) |  |
| **ICU Length of Stay** |  |  |  |  |  |  |  |  |  |  |  |  |
| Median [Q1, Q3] | 0 [0, 1] | 0 [0, 2] | 0.032* | 3 [2, 5] | 2 [2, 3] | 0.076 | 3 [2, 5] | 3 [2, 6] | 0.396 | 1 [0, 2] | 2 [1, 4] | <0.001* |
| Missing | 468 (49.7%) | 86 (45.0%) |  | 1170 (88.3%) | 360 (88.0%) |  | 604 (81.4%) | 165 (79.7%) |  | 836 (80.9%) | 233 (82.3%) |  |
| **Emergency Department Disposition** |  |  |  |  |  |  |  |  |  |  |  |  |
| Died | 3 (0.3%) | 0 (0%) | 0.018* | 3 (0.2%) | 0 (0%) | 0.658 | 4 (0.5%) | 2 (1.0%) | 0.519 | 2 (0.2%) | 0 (0%) | 0.822 |
| Discharged | 451 (47.9%) | 67 (35.1%) |  | 330 (24.9%) | 106 (25.9%) |  | 81 (10.9%) | 16 (7.7%) |  | 82 (7.9%) | 26 (9.2%) |  |
| Floor | 248 (26.3%) | 61 (31.9%) |  | 716 (54.0%) | 205 (50.1%) |  | 480 (64.7%) | 135 (65.2%) |  | 668 (64.7%) | 176 (62.2%) |  |
| ICU | 166 (17.6%) | 40 (20.9%) |  | 120 (9.1%) | 41 (10.0%) |  | 107 (14.4%) | 37 (17.9%) |  | 124 (12.0%) | 39 (13.8%) |  |
| Operating room | 73 (7.7%) | 22 (11.5%) |  | 151 (11.4%) | 54 (13.2%) |  | 64 (8.6%) | 17 (8.2%) |  | 153 (14.8%) | 42 (14.8%) |  |
| Transfered | 1 (0.1%) | 1 (0.5%) |  | 4 (0.3%) | 2 (0.5%) |  | 3 (0.4%) | 0 (0%) |  | 0 (0%) | 0 (0%) |  |
| Missing | 0 (0%) | 0 (0%) |  | 1 (0.1%) | 1 (0.2%) |  | 3 (0.4%) | 0 (0%) |  | 4 (0.4%) | 0 (0%) |  |
| **Hospital Discharge Disposition** |  |  |  |  |  |  |  |  |  |  |  |  |
| Died | 6 (0.6%) | 1 (0.5%) | 0.608 | 17 (1.3%) | 5 (1.2%) | 0.463 | 6 (0.8%) | 5 (2.4%) | 0.322 | 8 (0.8%) | 3 (1.1%) | 0.473 |
| Discharged | 722 (76.6%) | 123 (64.4%) |  | 1282 (96.8%) | 400 (97.8%) |  | 633 (85.3%) | 190 (91.8%) |  | 917 (88.8%) | 245 (86.6%) |  |
| Rehab/skilled nursing | 13 (1.4%) | 4 (2.1%) |  | 26 (2.0%) | 4 (1.0%) |  | 17 (2.3%) | 4 (1.9%) |  | 14 (1.4%) | 6 (2.1%) |  |
| Transferred | 5 (0.5%) | 1 (0.5%) |  | 0 (0%) | 0 (0%) |  | 1 (0.1%) | 0 (0%) |  | 0 (0%) | 0 (0%) |  |
| Missing | 196 (20.8%) | 62 (32.5%) |  | 0 (0%) | 0 (0%) |  | 85 (11.5%) | 8 (3.9%) |  | 94 (9.1%) | 29 (10.2%) |  |
| **Outcome** |  |  |  |  |  |  |  |  |  |  |  |  |
| Alive | 935 (99.3%) | 184 (96.3%) | 1 | 1305 (98.5%) | 404 (98.8%) | 0.822 | 732 (98.7%) | 202 (97.6%) | 0.339 | 1018 (98.5%) | 279 (98.6%) | 1 |
| Dead | 7 (0.7%) | 1 (0.5%) |  | 20 (1.5%) | 5 (1.2%) |  | 10 (1.3%) | 5 (2.4%) |  | 11 (1.1%) | 3 (1.1%) |  |
| Missing | 0 (0%) | 6 (3.1%) |  | 0 (0%) | 0 (0%) |  | 0 (0%) | 0 (0%) |  | 4 (0.4%) | 1 (0.4%) |  |
| **Admitting Service** |  |  |  |  |  |  |  |  |  |  |  |  |
| Critical care | 1 (0.1%) | 1 (0.5%) | 0.313 | 81 (6.1%) | 17 (4.2%) | 0.012* | 58 (7.8%) | 16 (7.7%) | 0.074 | 4 (0.4%) | 0 (0%) | 0.010* |
| Face | 139 (14.8%) | 36 (18.8%) |  | 33 (2.5%) | 9 (2.2%) |  | 5 (0.7%) | 1 (0.5%) |  | 354 (34.3%) | 70 (24.7%) |  |
| General surgery | 251 (26.6%) | 63 (33.0%) |  | 307 (23.2%) | 101 (24.7%) |  | 285 (38.4%) | 99 (47.8%) |  | 537 (52.0%) | 179 (63.3%) |  |
| GU | 2 (0.2%) | 1 (0.5%) |  | 11 (0.8%) | 3 (0.7%) |  | 4 (0.5%) | 0 (0%) |  | 1 (0.1%) | 0 (0%) |  |
| Hand | 0 (0%) | 0 (0%) |  | 0 (0%) | 0 (0%) |  | 5 (0.7%) | 2 (1.0%) |  | 6 (0.6%) | 0 (0%) |  |
| Neuro | 51 (5.4%) | 9 (4.7%) |  | 62 (4.7%) | 31 (7.6%) |  | 46 (6.2%) | 4 (1.9%) |  | 0 (0%) | 0 (0%) |  |
| Non-surgical | 23 (2.4%) | 10 (5.2%) |  | 54 (4.1%) | 8 (2.0%) |  | 42 (5.7%) | 13 (6.3%) |  | 30 (2.9%) | 5 (1.8%) |  |
| Orthopedic surgery | 9 (1.0%) | 1 (0.5%) |  | 388 (29.3%) | 122 (29.8%) |  | 194 (26.1%) | 47 (22.7%) |  | 0 (0%) | 0 (0%) |  |
| Plastic surgery | 11 (1.2%) | 2 (1.0%) |  | 10 (0.8%) | 10 (2.4%) |  | 16 (2.2%) | 9 (4.3%) |  | 11 (1.1%) | 1 (0.4%) |  |
| Vascular | 0 (0%) | 1 (0.5%) |  | 0 (0%) | 0 (0%) |  | 1 (0.1%) | 0 (0%) |  | 0 (0%) | 0 (0%) |  |
| Missing | 455 (48.3%) | 67 (35.1%) |  | 379 (28.6%) | 108 (26.4%) |  | 86 (11.6%) | 16 (7.7%) |  | 90 (8.7%) | 28 (9.9%) |  |
| * denotes statistically significant difference between 2020 and the historical average | | | | | | | | | | | | |
